# Acute Biliary Pancreatitis in the First Trimester of Pregnancy Without Abdominal Pain, Associated With Vomiting: A Rare Atypical Clinical Case Report and a Mini-Review of the Literature

**DOI:** 10.7759/cureus.69732

**Published:** 2024-09-19

**Authors:** Efthymia Thanasa, Anna Thanasa, Ioannis-Rafail Antoniou, Alexandros Leroutsos, Vasileios Papadoulis, Gerasimos Kontogeorgis, Ioannis Paraoulakis, Ioannis Thanasas

**Affiliations:** 1 Department of Health Sciences, Medical School, Aristotle University of Thessaloniki, Thessaloniki, GRC; 2 Department of Obstetrics and Gynecology, General Hospital of Trikala, Trikala, GRC; 3 Department of Anesthesiology, General Hospital of Trikala, Trikala, GRC

**Keywords:** acute pancreatitis, case report, cholecystectomy, complications, drugs, magnetic resonance imaging, pregnancy, ultrasound, vomiting

## Abstract

Acute pancreatitis is rare during pregnancy, with gallstone formation being the primary risk factor. This case report involves a 37-year-old pregnant woman, gravida 4, para 3, who presented to the Emergency Department of the General Hospital of Trikala at 13 weeks gestation, with vomiting for approximately 12 hours and no abdominal pain. A clinical examination by a surgical team, along with a history of cholelithiasis and supporting laboratory and ultrasound findings, led to the diagnosis of acute pancreatitis. The patient was carefully monitored and received immediate supportive treatment, including antibiotics. After a gradual improvement in clinical and laboratory findings, she was discharged on the sixth day of hospitalization, with the decision to postpone the cholecystectomy until after delivery, if possible. At 39 weeks of pregnancy, she delivered vaginally. One month later, she underwent a scheduled laparoscopic cholecystectomy. This case report describes a rare and atypical case of mild acute biliary pancreatitis in the first trimester of pregnancy, highlighting the management of the disease in pregnant women, which may vary depending on the severity of symptoms, potential complications, and gestational age.

## Introduction

Pancreatitis is a serious medical condition of the digestive tract that can be associated with pregnancy. Although its occurrence during pregnancy is uncommon, it presents a challenge in obstetric clinical practice [1]. Pancreatitis in pregnant women primarily presents acutely. Acute pancreatitis is rare in pregnancy, with an incidence ranging from one case per 1,000 to three cases per 5,000 pregnancies. It occurs most frequently in the second and third trimesters or the immediate postpartum period [2]. In more than 50% of cases, acute pancreatitis during pregnancy occurs in the third trimester [3]. It is estimated that over 90% of pregnant women who develop acute pancreatitis do so in the last two trimesters [4]. The condition is less common in the first trimester of pregnancy (as in our case) [5]. Additionally, it is more common in pregnant women of advanced maternal age, those who are obese, and multiparas. Ramin et al. reported that acute pancreatitis is much more common in multiparas compared to primiparas, with an estimated incidence of about 72% of all cases [6].

Acute pancreatitis is characterized by inflammation of the pancreas and the presence of activated peptic enzymes within the pancreas. Depending on the severity of symptoms and the occurrence of organ failure, acute pancreatitis is classified into three forms: mild, moderate, and severe [7]. In many cases, whether in pregnant or non-pregnant women, the etiology of acute pancreatitis cannot be clearly determined (idiopathic pancreatitis). However, due to the increased risk of gallstone formation during pregnancy, bile duct stone formation appears to be the main etiological factor responsible for the acute onset of the disease in pregnancy [5]. Additionally, hyperlipidemia, obesity, hyperparathyroidism, alcohol abuse, the use of various medications, hypertensive disorders of pregnancy, and major surgery are among the primary etiological factors implicated in the onset of acute pancreatitis in pregnant women [8,9].

This study describes a rare, atypical case of mild acute biliary pancreatitis in the first trimester of pregnancy, which was successfully treated conservatively. Emphasis is placed on the diagnostic and therapeutic approach for these patients, highlighting that the management of acute pancreatitis during pregnancy may vary depending on the severity of symptoms, potential complications, and gestational age.

## Case presentation

This case report concerns a 37-year-old pregnant woman, gravida 4 para 3, who presented to the Emergency Department of the General Hospital of Trikala during the 13th week of pregnancy with a 12-hour history of vomiting. Anorexia, nausea, and fever were not reported. Her medical history was notable for cholelithiasis and recent recurrent episodes of biliary colic prior to conception. Her body mass index (BMI) was 33. Obstetric follow-up indicated that the pregnancy had progressed normally up to this point, and there had been no episodes of biliary colic or cholecystitis during her previous pregnancies.

On clinical examination, the patient had a blood pressure of 110/70 mmHg and a heart rate of 91 beats per minute, both within normal limits. Mild epigastric tenderness was observed. The obstetric examination revealed no abnormal findings, and the first-trimester antenatal check-up results were normal. After admission, an upper abdominal ultrasound showed a liver of normal size and echogenicity. The gallbladder was enlarged and edematous, with a gallstone and wall thickening (>3 mm). The pancreas appeared edematous with blunted borders but showed no space-occupying lesions. Laboratory tests (Table 1) strongly suggested acute pancreatitis. Unfortunately, magnetic resonance imaging was not available at our hospital.

**Table 1:** Laboratory tests of our patient during her hospitalization at the clinic Ht: Hematocrit; Hb: Hemoglobin; WBC: White Blood Cells; NEUT: Neutral; CRP: C-reactive Protein; Glu: Glucose; TBIL: Total bilirubin; DBIL: Direct Bilirubin; IDBIL: Indirect Bilirubin; SGOT: Serum Glutamic Oxaloacetic Transaminase; SGPT: Serum Glutamate Pyruvate Transaminase; ALP: Alkaline Phosphatase; AMY: Amylase; LPS: Lipase; Chol: Cholesterol; Trig: Triglycerides

Laboratory tests	Day of admission to the clinic	Forty-eight hours of hospitalization	Seventy-two hours of hospitalization	Six days of hospitalization	Normal laboratory values
Ht	34.3%	32.9%	33.1%	35.6%	37.7 – 49.7%
Hb	11.1 gr/dl	10.5 gr/dl	10.7 gr/dl	11.6 gr/dl	11.8 – 17.8 gr/dl
WBC	17.4x10^3^/ml	15.1x10^3^/ml	12.2x10^3^/ml	8.6x10^3^/ml	4 – 10.8 x10^3^/ml
NEUT	93%	91%	82%	71%	40 – 75%
CRP	5.1 mg/dl	7.3 mg/dl	5.6 mg/dl	0.8 mg/dl	0.5 mg/dl
Glu	127 mg/dl	105 mg/dl	85 mg/dl	81 mg/dl	75 – 115 mg/dl
TBIL	1.35 mg/dl	1.47 mg/dl	1.42 mg/dl	0.97 mg/dl	0 – 1.2 mg/dl
DBIL	1.13 mg/dl	1.14 mg/dl	1.21 mg/dl	0.47 mg/dl	0 – 0.5 mg/dl
ΙDBIL	0.79 mg/dl	0.79 mg/dl	0.71 mg/dl	0.61 mg/dl	0 – 0.7 mg/dl
SGOT	65 IU/L	64 IU/L	64 IU/L	32 IU/L	5 – 33 IU/L
SGPT	69 IU/L	69 IU/L	68 IU/L	35 IU/L	10 – 37 IU/L
AMY	1224 U/L	1278 U/L	980 U/L	107 U/L	30 – 118 U/L
LPS	441 U/L	478 U/L	321 U/L	148 U/L	0 – 160 U/L
Chol	251 mg/dl	245 mg/dl	240 mg/dl	237 mg/dl	< 200 mg/dl
Trig	265 mg/dl	250 mg/dl	242 mg/dl	241 mg/dl	<150 mg/dl

**Figure 1:**
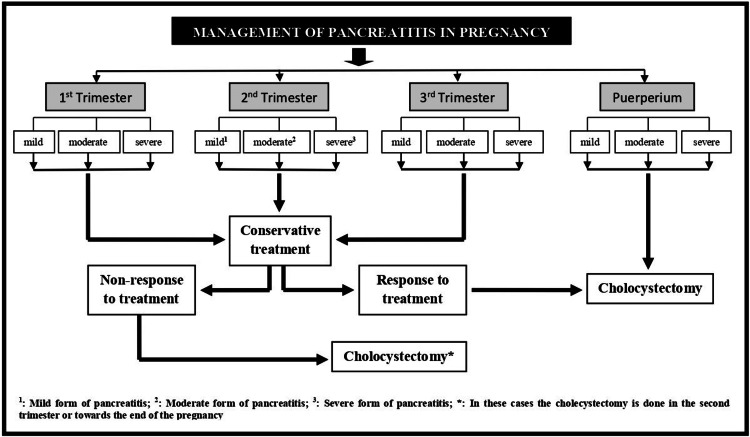
Schematic illustration depicting the management of pregnant women with acute pancreatitis The approach to managing acute biliary pancreatitis during pregnancy varies depending on the severity of symptoms, response to conservative treatment, and the gestational age (this image was created by the authors).

A team of consultant surgeons, based on clinical examination, laboratory results, and ultrasound findings, confirmed the diagnosis of acute biliary pancreatitis. The patient was closely monitored and treated with a second-generation cephalosporin and metronidazole. She was discharged from the clinic in good overall health on the sixth day of hospitalization, with instructions to attend regular follow-up appointments at the outpatient obstetric clinic. Due to the mild course of the disease, it was decided to postpone the cholecystectomy until later in pregnancy (Figure 1).

The patient eventually delivered vaginally at 39 weeks following spontaneous rupture of the fetal membranes. One month postpartum, she underwent a scheduled laparoscopic cholecystectomy.

## Discussion

Diagnosing acute pancreatitis during pregnancy can be challenging due to physiological changes in anatomical relationships caused by the gradual increase in uterine size, which complicates physical examination. This examination should be conducted with great care [10]. The predominant symptom of acute pancreatitis is epigastric or abdominal pain radiating to the back, corresponding to the location of the pancreas in the retroperitoneal space. This symptom is observed in the majority of patients (80%-95%). A smaller percentage of pregnant patients (40%-80%) also experience nausea and vomiting along with abdominal pain [11]. In extremely rare and atypical cases, vomiting with mild pain in the epigastric region or even without pain may be the primary symptom [12]. In such patients, particularly those in the first trimester of pregnancy (as in our case), the absence of epigastric pain can create significant challenges in differentiating acute pancreatitis from pregnancy-related nausea and vomiting. Additionally, fever, abdominal distension, tachycardia, tachypnea, dyspnea, orthostatic hypotension, and/or oliguria are uncommon but important clinical findings for diagnosing the disease [11].

The diagnostic criteria for acute pancreatitis include abdominal or epigastric pain radiating to the back, elevated serum amylase or lipase levels, and imaging findings of the liver, bile ducts, and pancreas. The presence of at least two of these three criteria confirms the diagnosis [7]. Testing for serum pancreatic enzymes plays a crucial role in diagnosing acute pancreatitis in pregnant women. Elevated levels of amylase (three or more times above the upper normal limit) and serum lipase have a diagnostic sensitivity estimated to reach approximately 94% of cases. Serum amylase typically increases within the first 12 hours after the onset of acute pancreatitis and peaks within 48 to 72 hours [13]. Additionally, laboratory testing in pregnant women with suspected pancreatitis should include a complete blood count, red blood cell sedimentation rate, C-reactive protein, and the quantification of other tests such as electrolytes, urea, creatinine, glucose, bilirubin, transaminases, cholesterol, and triglycerides. It is important to note that in pregnant women with acute pancreatitis caused by severe hypertriglyceridemia, serum amylase and lipase levels may be falsely normal [11].

In many cases, an increase in pancreatic enzymes may be a non-specific diagnostic finding. The elevation of pancreatic enzymes, when accompanied by severe abdominal pain, nausea, and vomiting, as seen in acute pancreatitis, should be considered in the differential diagnosis of other pathological conditions with obstetric or non-obstetric origins. These conditions include acute fatty liver of pregnancy, hemolysis, elevated liver enzymes and low platelets (HELLP) syndrome, placental abruption, uterine rupture, perforated gastric or duodenal ulcer, intestinal obstruction, acute cholecystitis, acute mesenteric ischemia, and acute urinary tract inflammation [14]. Additionally, acute appendicitis, particularly in the third trimester of pregnancy, presents a significant challenge in differential diagnosis. The characteristic progressive upward, rightward, and backward displacement of the cecum and appendix along the lateral abdominal wall due to the increasing uterine size can obscure palpation of the typical signs of appendicitis, making it difficult to distinguish from acute pancreatitis in pregnant women [15]. In our pregnant patient, the clinical diagnosis of acute pancreatitis was particularly challenging due to the absence of typical epigastric pain. The diagnosis was made based on elevated serum amylase and lipase levels, along with ultrasound imaging of the biliary tract and pancreas. Unfortunately, magnetic resonance imaging was not available at our hospital.

Among modern imaging modalities, ultrasound, particularly transabdominal and endoscopic ultrasound, is the most common, valuable, and safest test for diagnosing and addressing acute pancreatitis of cholelithiasis etiology in pregnant women. Transabdominal ultrasound can detect gallstones, inflammatory thickening of the gallbladder wall, pancreatic edema with blurred borders, peritoneal or peripancreatic fluid collections, and even necrosis of the pancreatic parenchyma with abscess or pseudocyst formation. These findings are indicative of acute inflammation of the gallbladder wall and pancreas [16]. Additionally, endoscopic ultrasound performed under anesthesia, without the use of radiation, can help avoid unnecessary invasive procedures like endoscopic retrograde cholangiopancreatography (ERCP) in pregnant women [17]. While ERCP is effective for detecting and removing gallstones, there is significant concern about its safety during pregnancy due to the use of ionizing radiation [18]. Magnetic resonance imaging is also considered a relatively safe method for diagnosing acute pancreatitis during pregnancy [17]. In contrast, the use of computed tomography during pregnancy is limited due to the exposure to ionizing radiation [19]. In our patient, transabdominal ultrasound, combined with clinical and laboratory findings, allowed for a prompt and accurate diagnosis of the disease, facilitating timely and effective treatment.

The management of pregnant women with acute pancreatitis depends on the severity of symptoms, the presence of complications, and the gestational age (Figure 1). Effective management requires the coordinated efforts of an obstetrician-gynecologist, pediatrician, gastroenterologist, anesthesiologist, and general surgeon to ensure the well-being of the fetus, newborn, and mother [20]. In most cases, conservative treatment is the safest and most effective approach to preventing serious complications. This includes ensuring adequate hydration, correcting electrolyte imbalances, and safely administering medications such as spasmolytics, analgesics, non-steroidal anti-inflammatory drugs, and antibiotics [21,22]. Antibiotics should be used when acute pancreatitis is associated with infectious causes, with ampicillin-sulbactam and piperacillin-tazobactam being safe options [23]. In cases where the disease is related to a hyperlipidemic profile (hypercholesterolemia and/or hypertriglyceridemia), low-fat parenteral nutrition may play a significant role in the conservative management of the condition [24].

The greatest challenge in managing acute pancreatitis during pregnancy is deciding whether to proceed with surgical treatment. Most experts suggest that in uncomplicated cases, conservative management is appropriate, with any invasive procedures being postponed until after delivery. If surgery cannot be delayed for an extended period, the second trimester is preferred for performing the procedure via a laparoscopic approach [25]. An indication for immediate cholecystectomy may arise when acute pancreatitis is secondary to cholelithiasis, typically after the acute inflammatory process has resolved [26]. Furthermore, severe hemorrhagic pancreatitis, pancreatic abscesses, and ruptured pancreatic pseudocysts are complicated forms of the disease, where immediate prenatal surgical intervention is expected to significantly benefit both the course of the disease and the pregnancy's uneventful progression [27,28]. In our patient, the mild form of the disease and the positive response to conservative treatment allowed the pregnancy to continue, with surgery scheduled for after delivery. A laparoscopic cholecystectomy was performed one month postpartum.

The prognosis of acute pancreatitis in pregnant women depends on several factors, including the severity of symptoms, the presence of complications, gestational age, and the timeliness of diagnosis, which enables early and effective treatment. The consequences of acute pancreatitis can be severe and even life-threatening for both the mother and fetus. Common complications include fetal demise, preterm delivery, adverse pregnancy outcomes, and neonatal death [29,30]. However, the widespread adoption of modern diagnostic and therapeutic approaches in recent years has contributed to a significant reduction in maternal and perinatal morbidity and mortality rates [31].

## Conclusions

Acute pancreatitis in pregnancy is a rare but potentially serious complication for the mother, fetus, and newborn. Even rarer is the atypical onset of the disease without typical abdominal pain. Prompt diagnosis is crucial. The management of pregnant women with acute pancreatitis may vary from case to case. Conservative treatment is generally the first step in the therapeutic approach, regardless of the severity of symptoms or gestational age. Antenatal surgical treatment of acute pancreatitis is indicated in selected cases.
